# Inactivation/deficiency of DHODH induces cell cycle arrest and programed cell death in melanoma

**DOI:** 10.18632/oncotarget.19379

**Published:** 2017-07-19

**Authors:** Lichao Liu, Zhen Dong, Qian Lei, Jie Yang, Huanrong Hu, Qian Li, Yacong Ji, Leiyang Guo, Yanli Zhang, Yaling Liu, Hongjuan Cui

**Affiliations:** ^1^ Department of Dermatology, The Third Hospital of Hebei Medical University, Shijiazhuang, 050000, China; ^2^ State Key Laboratory of Silkworm Genome Biology, Southwest University, Chongqing, 400715, China

**Keywords:** dihydroorotate dehydrogenase (DHODH), leflunomide, autophagy, BCL-2, melanoma

## Abstract

Malignant melanoma (MM) is one of the most malignant tumors and has a very poor prognosis. However, there are no effective drugs to treat this disease. As a kind of iron flavin dependent enzyme, dihydroorotate dehydrogenase (DHODH, EC 1.3.3.1) is the fourth and a key enzyme in the *de novo* biosynthesis of pyrimidines. Herein, we found that DHODH inactivation/deficiency inhibited melanoma cell proliferation, induced cell cycle arrest at S phase and lead to autophagy in human melanoma cells. Meanwhile, leflunomide treatment induced cell apoptosis and deficiency of DHODH sensitized cells to drug-induced apoptosis in BCL-2 deficient melanoma cells, while not in BCL-2 abundant melanoma cells. Then we found that BCL-2 could rescue apoptosis induced by DHODH inactivation/deficiency. Moreover, BCL-2 also showed to promote cell cycle arrest and to inhibit autophagy induced by leflunomide. To explore the mechanisms underlying autophagy induced by DHODH inhibition, we found that AMPK-Ulk1 axis was activated in this process. Besides, JNK was phosphorylated and activated to phosphorylate BCL-2, which abrogated the interaction between BCL-2 and Beclin1 and then abolished autophagy. Our findings provided evidences for the potential of DHODH used as a drug target for melanoma treatment.

## INTRODUCTION

As an aggressive and usually fatal malignancy, malignant melanoma (MM) is one of the fastest rising cancer and has a poor prognosis [1]. At present, the main treatment of melanoma is surgical resection, but only patients in early stage can be cured [2]. Once it has progressed to the metastatic stage, it remains an incurable disease, with a 5-year survival rate of 16% [3]. Until now, most chemotherapeutics and immunotherapeutics as well as radiotherapeutics have failed to increase survival rates of patients with malignant melanoma [4, 5]. Therefore, investigating some novel target therapy with high efficiency for malignant melanoma are problems to be solved.

Compared with normal cells, there are significant differences of metabolism within tumor cells [6]. Pyrimidine bases are elementary precursors used in nucleic acids biosynthesis, and are considered to be important for cellular metabolism, especially in rapid growth cells, such as cancer cells. Therefore, pyrimidine bases are suggested to be ideal targets for cancer treatment. As a kind of iron flavin dependent enzyme, dihydroorotate dehydrogenase (DHODH, EC 1.3.3.1), which is localized at the inner mitochondrial membrane, is the fourth and a key enzyme in the *de novo* biosynthesis of pyrimidines [7]. DHODH catalyzes oxidation of dihydroorotate to orotate, which is precursors of uridines and cytidine nucleosides [8]. Recently, DHODH was reported to play essential roles during tumorigenesis and cancer development [9–11]. These evidences indicated that DHODH might be a potential target for drug intervention in cancer treatment.

Early in 1959, the anti-proliferative effect of DHODH inhibitors was applied in tumor cells [12]. During last decades, researchers had discovered multiple DHODH inhibitors, such as leflunomide, brequinar, teriflunomide (A77 1726), benzimidazole and so on [13, 14]. As classic DHODH inhibitors, leflunomide and its active metabolite A77 1726 have been demonstrated to suppress cell proliferation or to induce cell death in various tumors [15–17]. Importantly, DHODH inhibition by leflunomide induced a significant decrease in melanoma growth both *in vitro* and *in vivo* studies [18]. Several other studies also showed that teriflunomide could suppress growth of melanoma cells [16, 19]. However, the mechanisms underlying remained to be further explored.

In this paper, we confirmed the function of leflunomide in human melanoma cells. Our studies put forward that DHODH inhibition by leflunomide or shRNA knockdown suppressed tumor growth and induced apoptosis and autophagy in melanoma cells. Besides, we also explored the molecular mechanisms underlying. Our findings provided evidences for the potential of therapeutic leflunomide using as a novel agent for melanoma treatment.

## RESULTS

### DHODH inhibitor leflunomide inhibits cell proliferation and induces cell cycle arrest at S phase in melanoma cells

**Figure 1:**
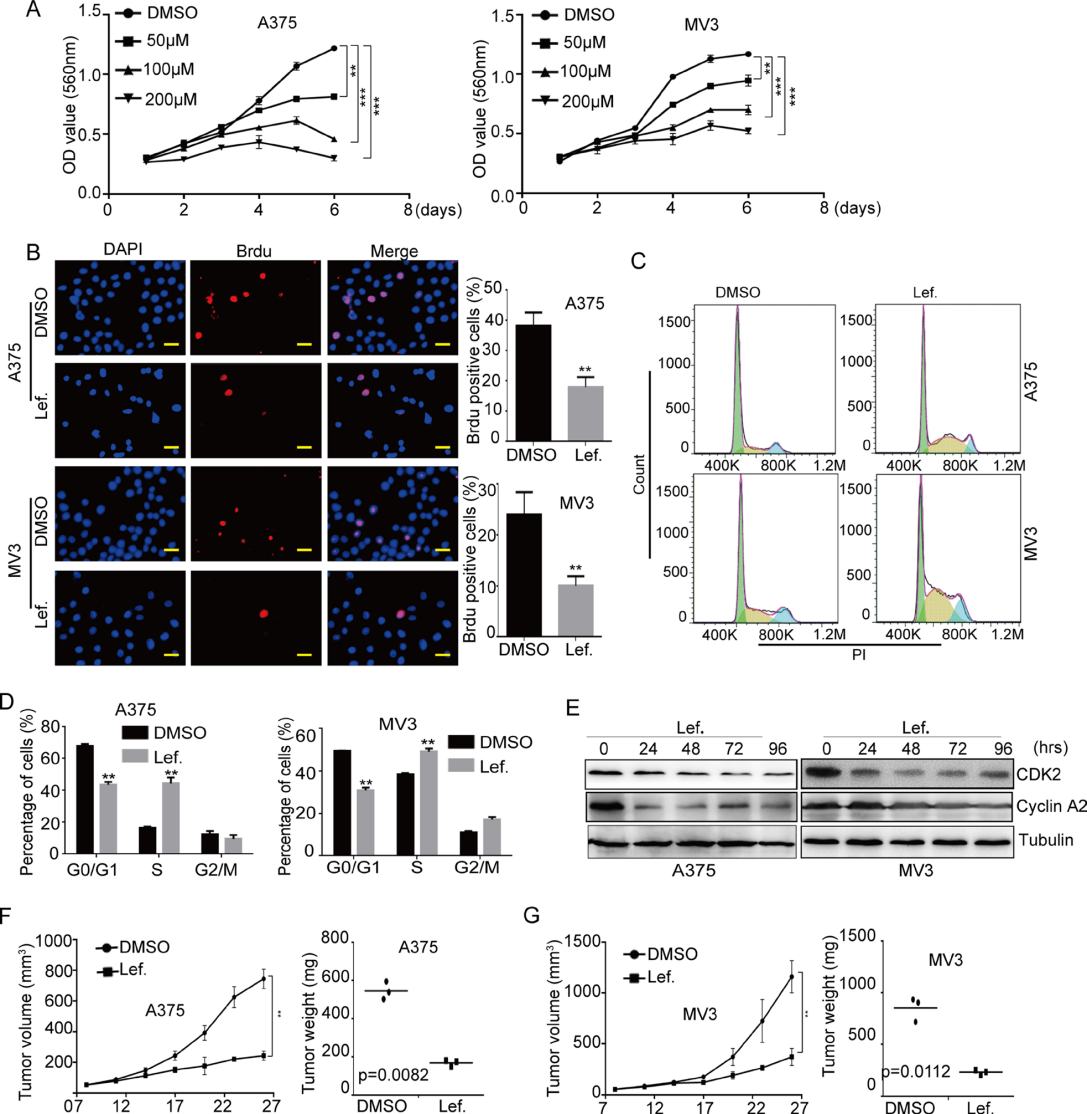
DHODH inhibitor leflunomide inhibits cell proliferation and induces cell cycle arrest at S phase in melanoma cells (**A**) Cell growth was tested by the MTT assay in A375 and MV3 cells after treated DMSO or 50, 100, 200 μM leflunomide (Lef.) for 1–6 days. (**B**) Image and quantification of A375 and MV3 cells positive for Brdu staining after treating with DMSO or 100 μM leflunomide for 72 h, Scale bar, 20 μm. (**C** and **D**) The cell cycle of A375 and MV3 cells was analyzed by flow cytometry after treatment with DMSO or leflunomide. (**E**) Western blot assay was performed to assess the cell cycle-related protein levels in A375 and MV3 cells after treatment with leflunomide for 0, 24, 48, 72 and 96 hours. Tubulin was used as a loading control. (**F** and **G**) The six weeks old female nude mice (BALA/c) xenograft tumor after treatment with DMSO or 7.5 mg/kg leflunomide. Xenograft tumor volume and weight were analyzed. All data are shown as the mean ± SD, Student's *t*-test was carried out. ^**^p < 0.01, ^***^p < 0.001.

To explore the effect of DHODH inhibition by leflunomide, we detected cell growth and proliferation by cell counting method, MTT assay and Brdu assay in human melanoma A375 and MV3 cells after treatment of leflunomide. Under the microscope, cells dealt with different concentrations of leflunomide for 72 h, resulting in a significant reduction in the viable cell number in a dose-dependent manner (Supplementary Figure 1A and 1B). Then we implied MTT assay, and the results showed that cell proliferation was markedly decreased in 50 μM, 100 μM and 200 μM leflunomide-treated groups compared with DMSO-treated groups (Figure 1A). Brdu staining assay also showed that cells dealt with 100 μM leflunomide for 72 h resulted in a remarkable decrease in the percentage of Brdu positive cells, compared with control groups (Figure 1B). These results certified that leflunomide inhibited cell growth and proliferation in human melanoma cells.

Since cell proliferation is usually regulated by cell cycle, we analyzed cell cycle of A375 and MV3 by flow cytometry to investigate whether leflunomide inhibited cell proliferation by inducing the cell cycle arrest. Cell cycle assessment showed that leflunomide-treated cells resulted into a distinct S phase arrest in A375 and MV3 cells, compared with the DMSO-treated group (Figure 1C and 1D). To confirm this result, we measured the expression of CDK2 and CyclinA2, which could promote cell cycle to go through the S phase. We found that the expression level of CDK2 and CyclinA2 were decreased in A375 and MV3 cells after leflunomide treatment in a time-course manner (Figure 1E). As DHODH inactivation blocked the catalyzation of dihydroorotate to orotate, which was precursor of uridine [8], we used 1 mM exogenous uridine to rescue nutritive deficiency of leflunomide-treated cells. The result showed that exogenous uridine could retrieve leflunomide induced cell cycle arrest (Supplementary Figure 1C and 1D). These results suggested that leflunomide induced cell cycle arrest by downregulating of CDK2-cyclin A2 complex in human melanoma.

Then soft agar assay was implemented to assess the effect of leflunomide in the self-renewal of melanoma cells. The result showed that A375 and MV3 dealt with leflunomide came into lesser and smaller colonies compared to DMSO group (Supplementary Figure 1E). To explore the effect of leflunomide in xenograft tumor, the melanoma A375 and MV3 cells were transplanted subcutaneously into female BALA/c nude mice, after seven days injected with 7.5 mg/kg leflunomide every three days for 12 days. At last the mice were sacrificed, and the formed tumors were removed. The results showed that leflunomide treatment significantly blocked tumor growth in both weight and size in nude mice (Figure 1F and 1G). These results indicated that leflunomide might be a potent target drug for melanoma treatment.

### Leflunomide induces apoptosis and autophagy in melanoma cells

**Figure 2:**
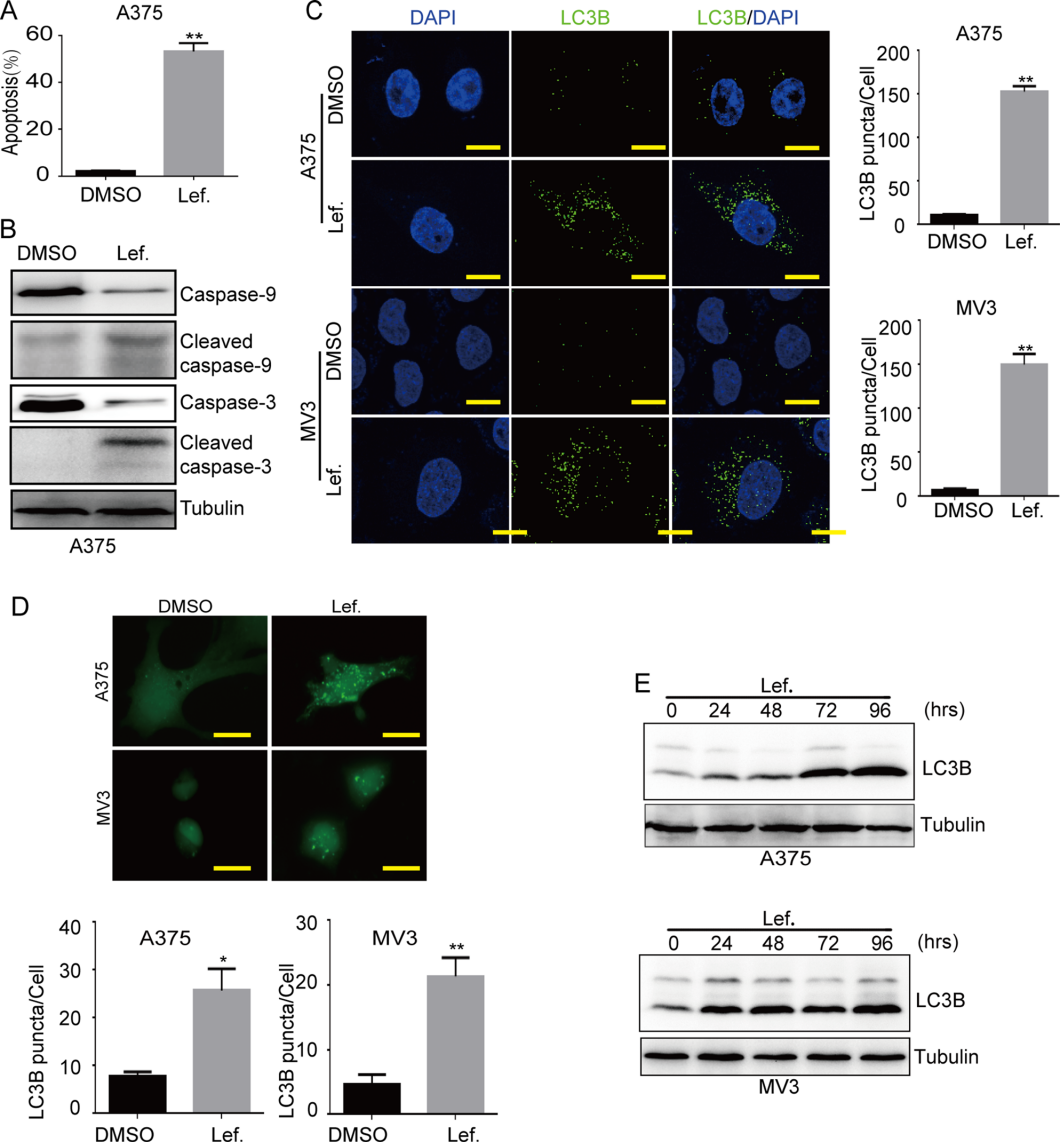
Leflunomide induces apoptosis and autophagy in melanoma cells (**A**) The rate of apoptosis of A375 cells was analyzed using flow cytometry after treated with DMSO or 100 μM leflunomide for 72 h. (**B**) Western blot assay was performed to assess the cell apoptosis-related protein levels in A375 cells after treatment with DMSO or leflunomide for 72 h, respectively. Tubulin was used as a loading control. (**C**) Immunofluorescence staining with a LC3B antibody was performed to confirm the induction of autophagy after treated with DMSO or 100 μM leflunomide for 72 h. Representative LC3B-positive cells are shown. Scale bars, 10 μm. (**D**) Cells transfected with the GFP-LC3B plasmid after treated with DMSO or 100 μmol/L leflunomide for 72 h were examined using fluorescence microscopy. Scale bars, 10 μm. The quantification of LC3B-positive puncta is presented as a histogram. (**E**) The level of autophagy was evaluated by LC3B expression, as determined using Western blot in A375 and MV3 cells after treated with 100 μmol/L leflunomide for 0, 24, 48, 72 and 96 hours. Tubulin was used as the loading control. All data are shown as the mean ± SD, Student's *t*-test was carried out. ^*^p < 0.05,^**^p < 0.01.

To detect whether viable cell number reduction after dealt with leflunomide was caused by apoptosis, cells were treated with 100 μM leflunomide or isometric DMSO for 72 h, then stained with AnnexinV-FITC/PI and analysed by flow cytometry. The results showed that leflunomide treatment brought out distinct apoptosis in A375 cells (Figure 2A). To further confirm it, we conducted Western blot assay and found that cleaved caspase-9 and cleaved caspase-3, which represented the caspase-dependent pathway of apoptosis, were increased compared with the control groups in A375 cells (Figure 2B and Supplementary Figure 2A). Besides, exogenous uridine also recovered leflunomide-induced apoptosis (Supplementary Figure 2B). These results showed that leflunomide induced apoptosis in A375 cells.

At the same time, we observed that there were more vesicles in the cells treated with leflunomide compared to the control groups under microscope. It was reported that hallmarks of autophagosome formation include the conversion of LC3A (LC3-I, 16 kd) to phosphatidylethanolamine (PE)-conjugated LC3B (LC3-II, 14 kd) during autophagosome closure [20]. In our further research, immunofluorescence was performed to detect the formation of LC3B, the results presented that leflunomide-treated cells showed more aggregations of LC3B puncta in both MV3 and A375 cells compared to the control groups (Figure 2C). Then, we transiently transfected melanoma cells with GFP-LC3B plasmids in both A375 and MV3 cells and observed the cells under a fluorescence microscope, and the results presented that leflunomide-treated cells showed more aggregations of GFP-LC3B puncta compared to DMSO-treated groups (Figure 2D). According to these results, we tested the expression of LC3B by Western blot assay in both A375 and MV3 cells, and the results showed a persistently increased conversion of LC3B in leflunomide-treated cells in a time-dependent manner (Figure 2E). In addition, exogenous uridine retrieved aggregations of LC3B puncta induced by leflunomide in both MV3 and A375 cells (Supplementary Figure 2C and 2D). To explore whether autophagy induced by leflunomide treatment promoted cell survival or cell death, we used two autophagy inhibitors, 3-MA (10 mM) and chloroquine (CQ, 25 μM) to block autophagy in A375 and MV3 cells. The results showed that cell viabilities were partly rescued after 3-MA or CQ treatment in leflunomide-treated groups (Supplementary Figure 2E). All together, these observations suggested that DHODH inactivation induced autophagy and contributed to cell death in human melanoma cells.

### DHODH knockdown suppresses cell proliferation and induces cell cycle arrest at S phase in melanoma cells

**Figure 3:**
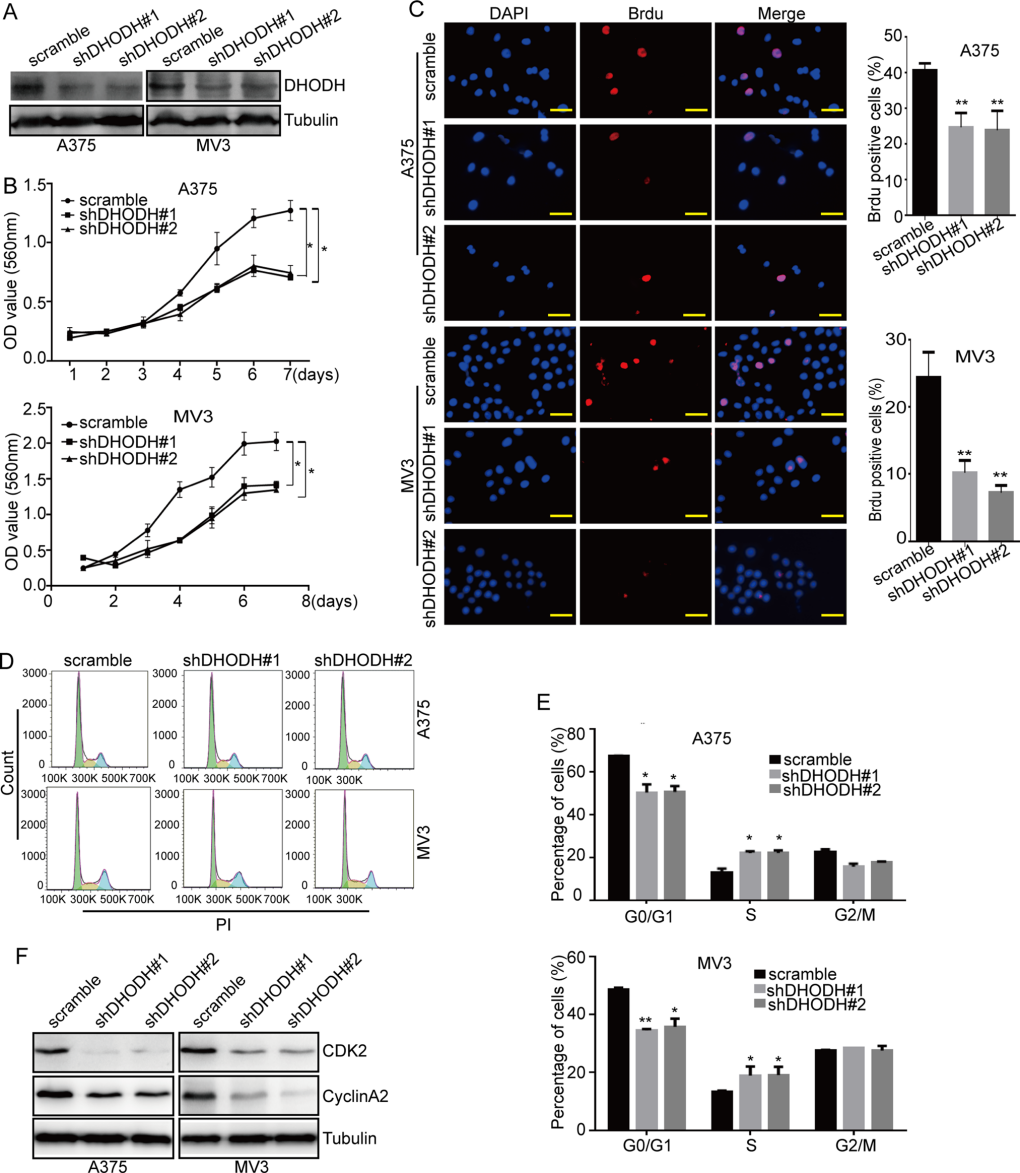
DHODH knockdown suppresses cell proliferation and induces cell cycle arrest at S phase in melanoma cells (**A**) Western blot assay was used to characterize the expression of DHODH in DHODH-knockdown A375 and MV3 cells, scramble was used as the control. The proteins of stable DHODH knockdown cell lines were collected after 48 hours normal culture. Tubulin was used as a loading control. (**B**) Cell growth was tested by the MTT assay in A375 and MV3 cells after DHODH knockdown. (**C**) Image and quantification of A375 and MV3 cells positive for Brdu staining after DHODH knockdown, Scale bar, 40 μm. (**D** and **E**) The cell cycle of A375 and MV3 cells was analyzed by flow cytometry after DHODH knockdown. (**F**) Western blot assay was performed to assess the cell cycle-related protein levels in A375 and MV3 cells after DHODH knockdown. The proteins of stable DHODH knockdown cell lines were collected after 48 hours normal culture. Tubulin was used as a loading control. All data are shown as the mean ± SD, Student's *t*-test was carried out. ^*^p < 0.05,^**^p < 0.01.

To address the importance of DHODH in cell proliferation and cell cycle, we used lentivirus carrying small hairpin RNA (shRNA) constructing against DHODH (shDHODH) or a scramble control to infect the human melanoma cells A375 and MV3, then cells were selected by puromycin. Western blot analysis showed that there was a significantly downregulation of DHODH in these two melanoma cells compared with scramble groups (Figure 3A). Then cell growth curvature was graphed according to the results of MTT assay after the knockdown of DHODH. The results showed that cell proliferation was distinctly reduced after DHODH deficiency compared with the scramble groups (Figure 3B). Brdu staining assay also showed that knockdown of DHODH resulted in a remarkable decrease in the percentage of Brdu positive cells compared with control groups (Figure 3C). These results indicated that DHODH was important for cell proliferation in human melanoma cells.

Then, we analyzed the cell cycle by flow cytometry to investigate whether cell proliferation inhibition induced by DHODH downregulation was related to cell cycle progression. As expected, DHODH-silenced cells resulted into a remarkable S phase arrest in human melanoma cells, compared with the control groups (Figure 3D and 3E). To affirm the results, we measured the expression of CDK2 and CyclinA2 by Western blot assay and found that the expression levels of CDK2 and CyclinA2 were decreased in DHODH-silenced cells (Figure 3F). In addition, exogenous uridine also retrieved leflunomide-induced cell cycle arrest (Supplementary Figure 3A and 3B). These results suggested that knockdown of DHODH induced cell cycle arrest in human melanoma cells by downregulating of the Cyclin A2-CDK2 complex. Then, the role of DHODH in colony formation was evaluated by soft agar assay. The results showed that DHODH-silenced cells gave rise to lesser and smaller colonies compared with scramble group (Supplementary Figure 3C). These results demonstrated that DHODH was essential for melanoma progression in human melanoma cells.

### DHODH downregulation sensitizes cells to drug-induced apoptosis and induces autophagy in melanoma cells

**Figure 4:**
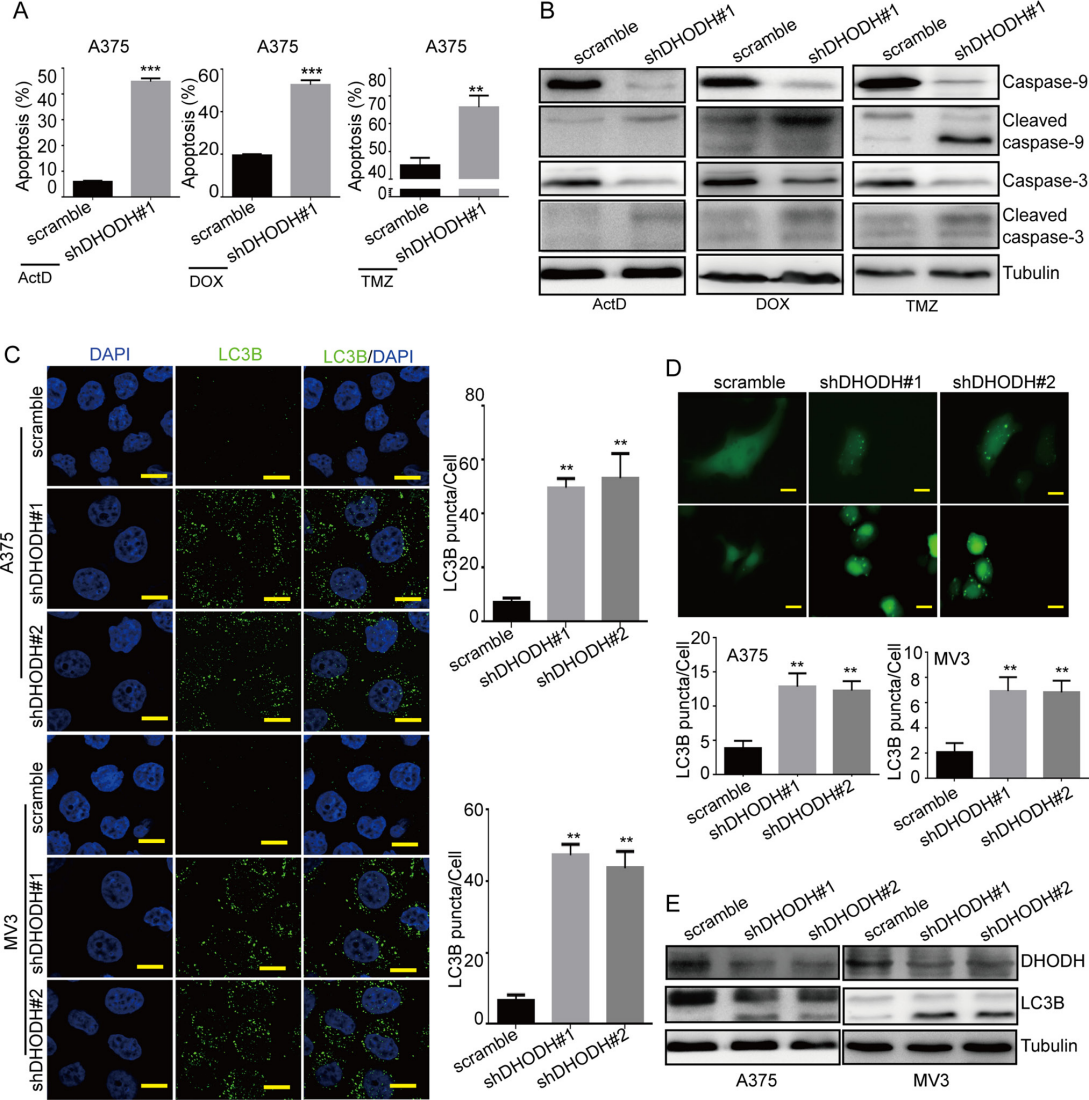
DHODH downregulation sensitizes drug-induced apoptosis and induces autophagy in melanoma cells (**A**) The rate of apoptosis was analyzed using flow cytometry in DHODH-knockdown A375 cells in the presence of 1 nM actinomycin D (ActD) for 24 h, 0.5 μM doxorubicin (DOX) for 12 h, or 400 μM temozolomide (TMZ) for 72 h in culture medium. (**B**) Western blot assay was performed to assess the cell apoptosis-related protein levels in DHODH-knockdown A375 cells in the presence of 1 nM actinomycin D (ActD) for 24 h, 0.5 μM doxorubicin (DOX) for 12 h, or 400 μM temozolomide (TMZ) for 72 h in culture medium. Tubulin was used as a loading control. (**C**) Immunofluorescence staining with a LC3B antibody was performed to confirm the induction of autophagy in A375 and MV3 cells after DHODH knockdown. Representative LC3B-positive cells are shown. Scale bars, 10 μm. (**D**) A375 and MV3 cells transfected with the GFP-LC3B plasmid after DHODH knockdown were examined using fluorescence microscopy. Scale bars, 10 μm. The quantification of LC3B-positive puncta is presented as a histogram. (**E**) The level of autophagy was evaluated by LC3B expression, as determined using Western blot in A375 and MV3 cells after DHODH knockdown. Tubulin was used as a loading control. All data are shown as the mean ± SD, Student's *t*-test was carried out. ^**^p < 0.01, ^***^p < 0.001.

To detect whether DHODH knockdown induced apoptosis in melanoma cells, we conducted flow cytometry to analyze apoptosis in DHODH knockdown cells. As showed in Supplementary Figure 4A, there was no significant apoptosis in A375 and MV3 cells after DHODH knockdown. So we supposed that DHODH might sensitize drug-induced apoptosis. Actinomycin D and doxorubicin were commonly used as apoptosis inducers, while temozolomide was used as a chemotherapy drug in melanoma treatment. To further explored whether DHODH downregulation affected apoptosis induced by these drugs, DHODH-silenced human melanoma cells were dealt with 1 nM actinomycin D for 24 h, 0.5 μM doxorubicin for 12 h, or 400 μM temozolomide for 72 h severally (DMSO as the control), then analyzed by flow cytometry. In comparison, both actinomycin D and doxorubicin were able to induce more pronounced cell apoptosis in A375 cells (Figure 4A). Accordingly, we tested the expression levels of cleaved caspase-9 and cleaved caspase-3 by Western blot assay and found that cleaved caspase-3 was increased in A375 cells after DHODH knockdown compared with the control group (Figure 4B). When exogenous uridine was added in medium, apoptosis induced by these drugs treatment was abrogated in DHODH-knockdown cells (Supplementary Figure 4B). These results showed that DHODH downregulation sensitized drug-induced apoptosis in A375 melanoma cells.

Furthermore, we evaluated the autophagy level in DHODH-silenced human melanoma cells. By immunofluorescent assay, we found that DHODH-silenced cells showed more puncta aggregations of LC3B, which was different from the diffuse LC3B observed in the control groups (Figure 4C and 4D). Then we detected the expression of LC3B and the result showed that LC3B expression was also increased in DHODH-knockdown cells than that in control groups (Figure 4E). Besides, increased LC3B puncta aggregations induced by DHODH deficiency could be rescued by exogenous uridine (Supplementary Figure 4C and 4D). In conclusions, these clues indicated that DHODH deficiency induced autophagy in A375 and MV3 melanoma cells.

### BCL-2 is a switch of apoptosis induced by DHODH inhibition

**Figure 5:**
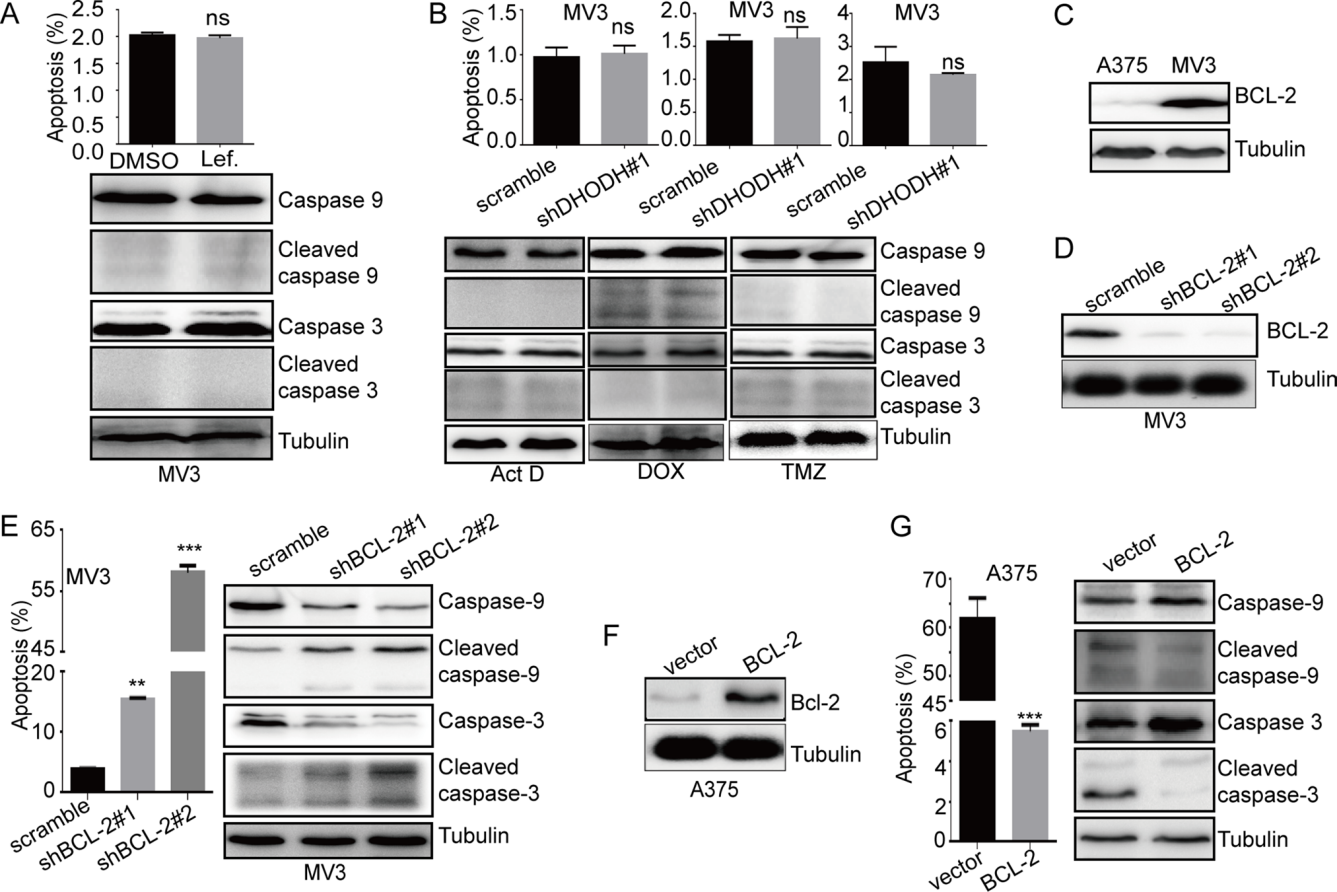
BCL-2 is a switch of apoptosis induced by DHODH inhibition (**A**) The rate of apoptosis of MV3 cells was analyzed using flow cytometry after treated with 100 μM leflunomide, and Western blot assay was performed to assess the cell apoptosis-related protein levels, respectively. (**B**) The rate of apoptosis of MV3 cells was analyzed by using flow cytometry after DHODH knockdown in the presence of 1nM actinomycin D (ActD) for 24 h, 0.5 μM doxorubicin (DOX) for 12 h, or 400 μM temozolomide (TMZ) for 72 h in medium. Western blot assay was performed to assess the cell apoptosis-related protein levels, respectively. (**C**) The expressiom of BCL-2 in human melanoma cells A375 and MV3 was measured by Western blot assay. (**D**) Western blot assay was performed to assess the protein levels of BCL-2 in BCL-2-knockdown MV3 cells. (**E**) The rate of apoptosis analyzed by flow cytomentry in BCL-2-knockdown MV3 cells after dealt with 100 μM leflunomide, and Western blot assay was performed to assess the cell apoptosis-related protein levels, respectively. (**F**) Western blot assay was performed to assess the protein levels of BCL-2 in BCL-2-overexpressed A375 cells. (**G**) The rate of apoptosis in BCL-2-overexpressed A375 cells after dealt with 100 μM leflunomide, and Western blot assay was performed to assess the cell apoptosis-related protein levels, respectively. All data are shown as the mean ± SD, Student's *t*-test was carried out. ^**^p < 0.01, ^***^p < 0.001, ns, no sense.

During our experiments, we found that there was no apoptosis in MV3, neither in leflunomide-treated group nor apoptosis inducers-treated group (Figure 5A and 5B). Accidently, we found that there was a higher expression of BCL-2 in MV3cells than that in A375 cells (Figure 5C). As a big family in mitochondria, BCL-2 protein family, which contained at least 16 members, played an important role in the modulation of intrinsic mitochondrial apoptosis pathway [21]. They were categorized into three functional groups: the pro-survival BCL-2-like family members, including BCL-2, MCL-2, BCL-X_L_, BFL1 and BCL-w; the multi-BH-domain pro-apoptotic members, such as BAX and BAK; and the pro-apoptotic BH3-only proteins, such as BIM, BID, BIK, PUMA, NOXA, BAD, HRK, BLK and BMF [22]. We conducted a qRT-PCR to detect the different expression of these members in MV3 and A375 cells, and the results showed that there was less *bcl-2* expression in A375 cells (Supplementary Figure 5A). These results indicated that BCL-2 might be a critical factor of apoptosis induced by DHODH inhibition in human melanoma cells.

To confirm this result, MV3 cells was infected with lentivirus which carrying small hairpin RNA (shRNA) constructing against BCL-2 (shBCL-2) or a scramble control, subsequently selected by puromycin. Western blot analysis showed that BCL-2 was significantly downregulated after knockdown by BCL-2 shRNAs in MV3 cells (Figure 5D). Then cell apoptosis was confirmed by flow cytometry assay in BCL-2-silenced MV3 cells, the result showed that BCL-2 knockdown didn't induce apoptosis (Supplementary Figure 5B). However, leflunomide treatment induced marked apoptosis in BCL-2-silenced MV3 cells contrasted with scramble groups (Figure 5E). Besides, we measured the expression of cleaved caspase-9 and cleaved caspase-3 and the result showed that the spliceosomes of caspase-9 and caspase-3 were increased in BCL2-silenced MV3 cell which dealt with leflunomide (Figure 5E). In addition, similar results also showed in MV3 cells treated with ABT, a specific BCL-2 inhibitor (Supplementary Figure 5C and 5D). At the same time, A375 cells were infected with lentivirus encoding BCL-2, subsequently selected by puromycin. Western blot analysis showed that BCL-2 was significantly upregulated in A375 cells (Figure 5F). Similarly, we conducted cell apoptosis by flow cytometry, and measured the expression of caspase-3 by Western blot assay. The results showed that overexpression of BCL-2 didn't affect apoptosis in normal culture condition (Supplementary Figure 5E), but rescued apoptosis induced by leflunomide in A375 cells (Figure 5G). In conclusions, these results indicated that BCL-2 was a switch of apoptosis induced by DHODH inhibition.

### DHODH inactivation/deficiency-induced cell cycle arrest and autophagy are correlated with BCL-2 expression

**Figure 6:**
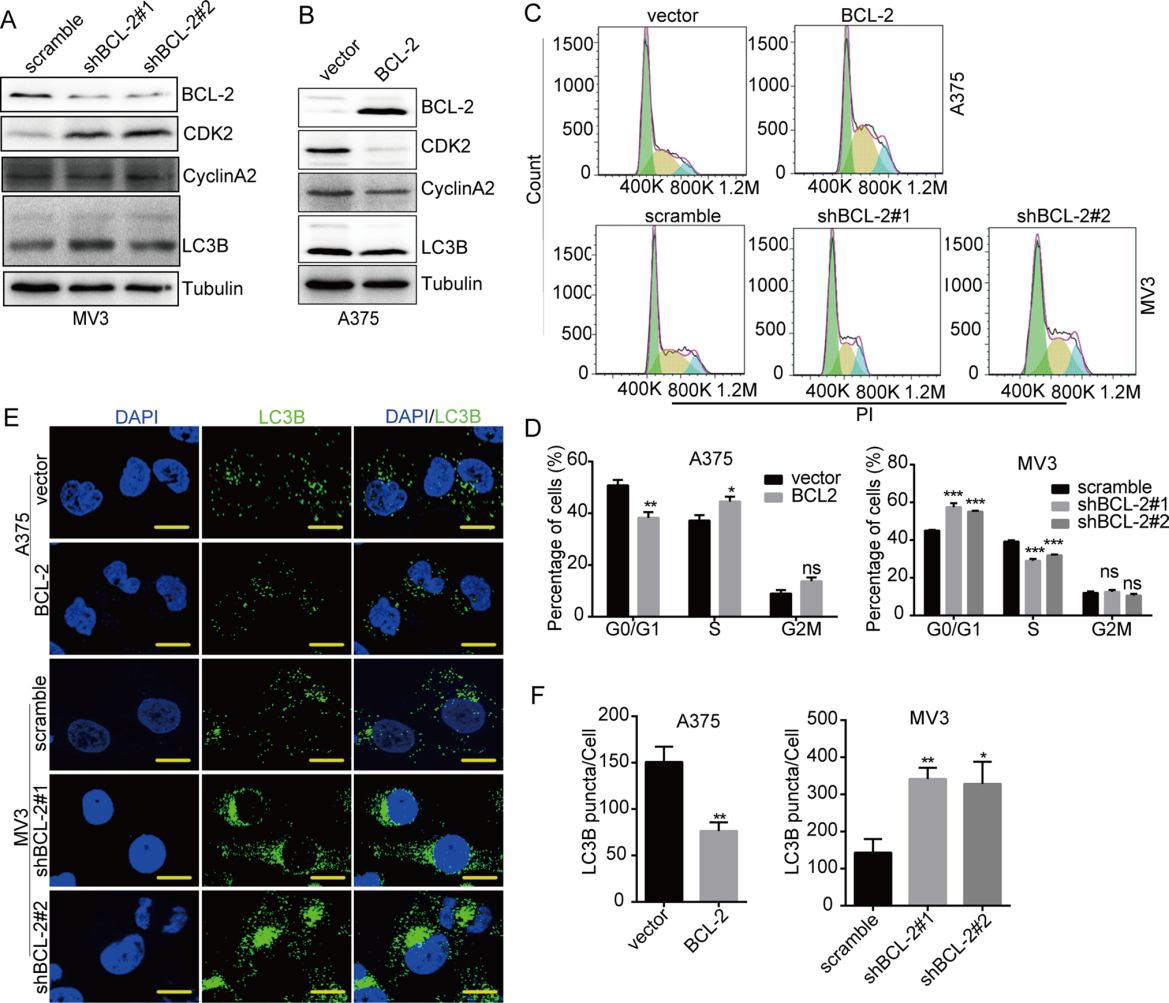
DHODH inhibition-induced cell cycle arrest and autophagy are regulated by BCL-2 (**A**) Western blot assay was performed to assess the protein levels of cell cycle-related proteins and LC3B in BCL-2 knockdown MV3 cells in the presence of 100 μM leflunomide in medium for 72 h. (**B**) Western blot assay was performed to assess the protein levels of cell cycle-related proteins and LC3B in BCL-2 overexpressed A375 cells in the presence of 100 μM leflunomide in medium for 72 h. (**C** and **D**) The cell cycle of BCL-2 overexpressed A375 and BCL-2 knockdown of MV3 cells was analyzed by flow cytometry in the presence of 100 μM leflunomide in medium for 72 h. (**E** and **F**) Immunofluorescence staining with a LC3B antibody was performed to confirm the induction of autophagy in BCL-2 overexpressed A375 and BCL-2 knockdown of MV3 cells in the presence of 100 μM leflunomide in medium for 72 h. Representative LC3B-positive cells are shown. Scale bars, 10 μm. All data are shown as the mean ± SD, Student's *t*-test was carried out. ^*^p < 0.05,^**^p < 0.01, ^***^p < 0.001, ns, no sense.

Recently, BCL-2 was shown to play essential roles during cell cycle and autophagy regulation [23–25]. So we supposed that BCL-2 might also play some roles in the regulation of cell cycle arrest and autophagy induced by DHODH inactivation/deficiency. Western blot showed that CDK2 was increased in BCL-2 knockdown MV3 cells, while decreased in BCL-2 overexpressed A375 cells, in the presence of 100 μM leflunomide (Figure 6A and 6B). Flow cytometry results also showed that cell cycle arrest at S phase was also aggravated in BCL-2 overexpressed A375 cells, while remitted in BCL-2 knockdown MV3 cells, in the presence of 100 μM leflunomide (Figure 6C and 6D). These results indicated that BCL-2 inhibited cell cycle progression in melanoma cells. In addition, LC3B expression increased in BCL-2 knockdown MV3 cells, while decreased in BCL-2 overexpressed A375 cells, in the presence of 100 μM leflunomide (Figure 6A and 6B). Besides, LC3B puncta observed under laser confocal microscope showed similar results (Figure 6E and 6F). These results indicated that BCL-2 was an autophagy inhibitor in leflunomide-treated melanoma cells. In summary, BCL-2 was a key regulator of autophagy and cell cycle arrest induced by DHODH inhibition in melanoma cells.

### DHODH inactivation/deficiency induces autophagy via AMPK-Ulk axis and BCL-2 phosphorylation

**Figure 7:**
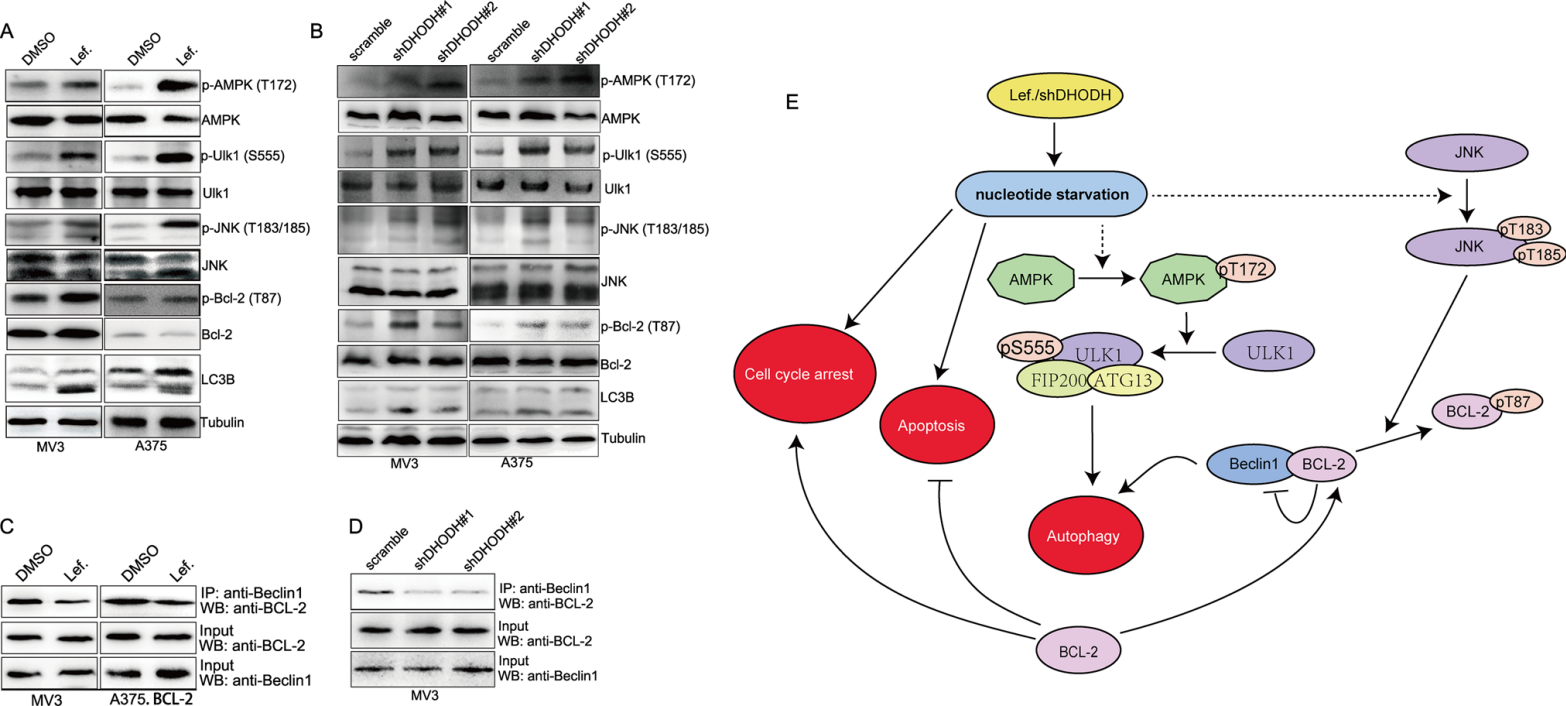
DHODH inhibition induces autophagy via AMPK-Ulk axis and BCL-2 phosphorylation (**A**) Western blot assay was performed to assess the protein levels of AMPK-Ulk axis and BCL-2 phosphorylation in MV3 and A375 cells treated with 100 μM leflunomide or DMSO for 72 h. (**B**) Western blot assay was performed to assess the protein levels of AMPK-Ulk axis and BCL-2 phosphorylation in MV3 and A375 cells after DHODH knockdown. (**C**) co-Immunoprecipitation assay was performed to detect the interaction between BCL-2 and Beclin1 in MV3 and BCL-2 overexpressed A375 cells treated with 100 μM leflunomide or DMSO for 72 h. (**D**) Co-Immunoprecipitation assay was performed to detect the interaction between BCL-2 and Beclin1 in MV3 cells after DHODH knockdown. (**E**) Overview of the mechanism of DHODH inhibition induced cell cycle arrest, apoptosis and autophagy. DHODH knockdown or leflunomide treatment induced nucleotide starvation in melanoma cells, and induced cell cycle arrest, apoptosis, and autophagy, which could be influenced by BCL-2 levels in cells. In this process, AMPK-Ulk axis was activated to induce autophagy and JNK and BCL-2 was phosphorylated to decrease the interaction between BCL-2 and Beclin1, thus induced autophagy.

It was reported that starvation could induce activation of AMPK, and the subsequent phosphorylation (Ser 555) and activation of Ulk1, and in turn the induction of autophagy [26–31]. So we want to detect whether AMPK-Ulk axis was essential during autophagy caused by DHODH inhibition-induced nucleotides starvation. As expected, AMPK was activated by phosphorylation of Thr 172 in MV3 and A375 cells after treated with 100 μM leflunomide for 72 hours (Figure 7A) or DHODH knockdown (Figure 7B). Besides, Ulk was inactivated by phosphorylation of Ser 555 in MV3 and A375 cells after treated with 100 μM leflunomide for 72 hours or DHODH knockdown (Figure 7A and 7B). These evidences indicated that AMPK-Ulk axis participated into DHODH inhibition-induced autophagy. It was reported that BCL-2 interacted with Beclin1 and inhibited its downstream activation in autophagy [32]. While phospho-JNK could phosphorylate BCL-2 and inhibit it to interact with Beclin1 [32]. So we detected whether BCL-2 was phosphorylated during this process. As expected, BCL-2 was further phosphorylated at Thr 87 in MV3 and A375 cells after treated with 100 μM leflunomide for 72 hours or DHODH knockdown (Figure 7A and 7B). Besides, the interaction between BCL-2 and Beclin1 was weakened after treated with 100 μM leflunomide for 72 hours or DHODH knockdown (Figure 7C and 7D). These evidences indicated that BCL-2 was also involved into DHODH inhibition-induced autophagy. In summary, DHODH inactivation/deficiency might induce autophagy through AMPK-Ulk axis and JNK-BCL-2 axis (Figure 7E).

## DISCUSSION

At present, melanoma is one of the most aggressive and drug-resistant human tumors. However, the genetic events that driving melanoma progression are less understood, and much efforts are required to identify driving factors and susceptible factors that could be pharmaceutical targets of melanoma. Since cancerous cells need more nutrient substance to produce new cells, metabolic signaling pathways are suggested to be essential during carcinogenesis [33]. As a rate-limiting enzyme of nucleotide metabolism pathway, DHODH seemed to contribute to malignant characteristics of various tumors, including multiple myeloma [16], chronic lymphocytic leukemia [34–37], neuroblastoma [38], colon cancer [9], prostate cancer [39], and so on.

Importantly, DHODH seemed to be a potential target in human malignant cutaneous tumors, especially melanoma. Expression level of DHODH was higher in precancerous cells and malignant cutaneous keratinocytes, compared to that in normal cutaneous cells [40]. Besides, DHODH inhibition by teriflunomide encouraged cytostatic and apoptotic effects in premalignant and malignant cutaneous keratinocytes [41]. Importantly, DHODH inhibition by leflunomide suppressed melanoma formation and growth in both zebrafish and mouse model [18]. However, the exact molecular mechanism of DHODH inhibition-induced cell proliferation suppression in melanoma was not fully elucidated.

Herein, we applied experiments to explore the function of DHODH in melanoma by using both a commonly used clinical DHODH inhibitor leflunomide and shRNAs of DHODH. As expected, DHODH inactivation/deficiency induced significant cell proliferation suppression in human A375 and MV3 melanoma cells (Figure 1A and 3B). Besides, Brdu positive cells were also reduced in these cells after DHODH inhibition (Figure 1B and 3C). Besides, tumor volume and weight was also reduced in A375 and MV3 melanoma xenograft mouse models (Figure 1F and 1G). These results consisted with previous reports [18], and showed that DHODH inhibition suppressed melanoma proliferation both *in vitro* and *in vivo*, indicating that DHODH might be a potential target in melanoma treatment.

As DHODH is one of the important rate-limiting enzyme during biological *de novo* synthesis of nucleotides, its inactivation/deficiency might reduce the abundance of nucleotides in the cells, thus affected cell cycle progression. Previous reports showed that A77 1726 induced G1 cell cycle arrest via modulation of cyclin D2 and pRb expression in myeloma cells [17]. Our results showed that DHODH inactivation/deficiency induced cell cycle arrest at S phase in A375 and MV3 cells (Figures 1C, 1D, 3D and 3E). In addition, CDK2 and CyclinA2, key regulators of S phase progression [42], were also dramatically reduced after DHODH inactivation/deficiency (Figures 1E and 3F). Consistently, Doscas ME et al. also found that A77 1726 accelerated cell cycle entry into the S phase through ERK1/2 activation in A375 cells and that pyrimidine nucleotide depletion halted cell cycle progression [16]. Our results further confirmed that DHODH inactivation/deficiency suppressed cell proliferation through S phase arrest.

Programmed cell death (PCD), including apoptosis, autophagy and programmed necrosis, is death of a cell mediated by intracellular programs. It decides the fate of cancer cells and its dysfunction provides an important clue to treat tumors [43]. Among them, apoptosis is the most common type of PCD, which characterized by nuclear fragmentation and pyknosis, chromatin condensation, as well as caspases activation, DNA breakdown and modifications of membrane surface that allow phagocytic cells to recognize and engulf the apoptotic cells [44]. Apoptosis is mainly triggered by two major mechanisms: one is extrinsic pathway, in which death ligands bind to death receptors; another is intrinsic mitochondrial pathway, in which cells are triggered to release cytochrome *c* from mitochondria to cytoplasm and caspase-9 is cleaved and auto-activated [45]. In both two apoptotic pathways, executioner caspase-3 is cleaved and activated to induce cell death [46].

In our results, DHODH inactivation by leflunomide also induced apoptosis in A375 melanoma cells, and both caspase-9 and caspase-3 were cleaved (Figure 2B), which indicating that DHODH inactivation induced intrinsic mitochondrial pathway of apoptosis in melanoma. However, DHODH knockdown couldn't induce significant apoptosis (Supplementary Figure 4A). These might be a result of a not so efficient knockdown by shRNAs. Because DHODH was an enzyme, a little expression was enough for a cell to use under normal culture condition, but might be not in stressful environments. Our results showed that DHODH knockdown sensitized A375 cells to apoptosis induced by extracellular stress, such as actinomycin D, doxorubicin, as well as temozolomide (Figure 4A and 4B). These results indicated that DHODH inhibitors combined with other chemotherapeutic drugs might be a novel therapeutic method for melanoma treatment.

Autophagy is a cellular catabolic process with degrading and recycling intracellular components in response to cell stress [47]. During this process, cytoplasmic components, such as organelles and cytosolic molecules, are engulfed by autophagosomes. Meanwhile, LC3-I is conjugated to phosphatidylethanolamine to form LC3-II, which is recruited to the membrane of autophagosomes [48]. Defects in the autophagy machinery are associated to the pathogenesis of many diseases including autoimmune, neurodegenerative, heart and liver disorders as well as cancer [49]. It was reported that autophagy played a dual role in cancer: on the one hand it could inhibit the occurrence and development of tumor, on the other hand it could also make cancer cells to adapt to adverse metabolic environment leading to the survival of tumor cells [50]. Our results also showed that DHODH inactivation/deficiency induced a persistently increased LC3B expression in A375 and MV3 melanoma cells in a time-course manner (Figure 2E and 4E). Besides, laser confocal microscopic images showed that LC3B puncta aggregations in cells were increased after DHODH inactivation/deficiency (Figure 2C and 4C). After transfected exogenous GFP-linked LC3B into cells, cells also showed more puncta aggregations of LC3B, which was different from the diffuse LC3B observed in the control groups (Figure 2D and 4D). In addition, autophagy inhibitor 3-MA and chloroquine partly rescued leflunomide-induced cell viability (Supplementary Figure 2E). These results indicated that autophagy might contribute to cell death induced by DHODH inhibition in melanoma cells.

To explore the mechanism underlying, we detected several essential regulators in autophagy pathway, including Erk1/2, DAPK1, p62 and Beclin1, the results showed that no significant expression changes of these proteins (Data not shown). However, we found that AMPK was phosphorylated at Thr 172, a prerequisite for AMPK activity, after DHODH inactivation/deficiency (Figure 7A and 7B). There were several mechanisms by which AMPK could promote autophagy. Historically, AMPK was an established negative regulator of the mTOR signaling cascade [51]. This could be accomplished by AMPK-mediated phosphorylation of the TSC complex which was a negative regulator of mTORC1 activation at the lysosome [52]. Alternatively, AMPK could directly phosphorylate the Raptor subunit of the mTORC1 complex, which induced 14-3-3 binding and inhibited mTORC1 target phosphorylation [53]. However, in 2010, Behrends et al. first discovered the interaction between AMPK and Ulk1/2 in a global proteomic analysis of the human autophagy network [54]. Subsequently, different groups confirmed the direct interaction between these two kinases [26–31]. Some of them additionally reported the AMPK-mediated phosphorylation of Ulk1. Phosphorylated Ulk activated itself and subsequently phosphorylated Beclin-1 on Ser 14, thereby enhancing the activity of the ATG14L-containing VPS34 complexes, thus induced autopghagy [55]. So we showed that Ser 555 phosphorylation of Ulk was increased after DHODH inhibition (Figure 7A and 7B). Our results indicated that AMPK-Ulk axis was essential in DHODH inhibition-induced autophagy (Figure 7E).

In addition, we found that DHODH inactivation/deficiency-induced cell cycle arrest, apoptosis and autophagy could be rescued by adding exogenous uridine in the medium (Supplementary Figures 1C, 1D, 2B, 2C, 2D, 3A, 3B, 4B,4C and 4D). These results indicated that DHODH inhibition-induced uridine deficiency contributed to cell cycle arrest, apoptosis and autophagy.

Interestingly, we found that DHODH inactivation/deficiency-induced apoptosis didn't occur in MV3 cells (Figure 5A and 5B). And we found that the expression of BCL-2 was much higher in MV3 cells than that in A375 cells (Figure 5C). Further qRT-PCR analysis also revealed that there was little *bcl-2* expression in A375 cells (Supplementary Figure 5C). As one of the core member of BCL-2 families, BCL-2 is localized to the inner mitochondrial membrane [56] or the plasma membrane [57] and is an essential anti-apoptotic factor in the intrinsic mitochondrial pathway of apoptosis [21]. BCL-2 could keep pro-apoptotic BAX and BAK in check, preventing them causing mitochondrial outer membrane permeabilization (MOMP) of apoptogenic factors such as cytochrome c into the cytoplasm [58]. Besides, BCL-2 was considered as an oncogene in multiple tumors, including melanoma, and contributed to cancer cell survival [59–61]. In addition, *bfl1* expression in A375 cells was extremely higher than MV3 cells (Supplementary Figure 5A). However, it was considered as an anti-apoptotic factor. So we supposed that BCL-2 was one of the most essential members for the survival of melanoma cells, and we knocked down BCL-2 in MV3 cells. The result showed that leflunomide induced remarkable apoptosis and activated caspase-9 and caspase-3 activities after BCL-2 knockdown (Figure 5D and 5E). In addition, BCL-2 overexpression retrieved leflunomide-induced apoptosis in A375 cells (Figure 5F and 5G). These results showed that BCL-2 was a switch of DHODH inactivation/deficiency-induced apoptosis.

It was reported that Bcl-2 proteins bound to Beclin1 through a BH3 domain and disrupt its autophagy function [24]. Our results showed that LC3B expression increased in BCL-2 knockdown MV3 cells, while decreased in BCL-2 overexpressed A375 cells (Figure 6A and 6B). Besides, LC3B puncta aggregations observed under laser confocal microscope showed similar results (Figure 6E and 6F). These results indicated that BCL-2 inhibited autophagy in melanoma cells after leflunomide treatment. Besides, the interaction between BCL-2 and Beclin1 was decreased after DHODH inhibition (Figure 7C and 7D). It was reported that nutrient starvation could activate JNK by its Thr 87 phosphorylation directly or through AMPK activiation, and phospho-JNK could phosphorylate BCL-2 and inhibit it to interact with Beclin1 [32]. Our results showed that Thr 87 phosphorylation of BCL-2 was increased in MV3 and A375 cells after DHODH inactivation/deficiency (Figure 7A and 7B). Our results provided an alternative underlying mechanism of DHODH inactivation/deficiency -induced autophagy.

It was reported that BCL-2 could inhibit cell cycle entry by facilitating G0 [23]. However, BCL2 didn't significantly affect growth rates under optimal conditions, but prolonged G1 in suboptimal conditions [62]. Our results showed that CDK2 was increased in BCL-2 knockdown MV3 cells, while decreased in BCL-2 overexpressed A375 cells (Figure 6A and 6B). The results of flow cytometry also showed that BCL-2 promoted cell cycle arrest in melanoma cells (Figure 6C and 6D). These results indicated that BCL-2 inhibited cell cycle in leflunomide-treated melanoma cells. Our results indicated that combination inhibition of BCL-2 and DHODH might be a new therapeutic regimen for malignant melanoma treatment. However, much more need to be considered in this therapeutic regimen because inhibiting BCL-2 might promote cell cycle progression.

In conclusions, our data showed that DHODH inhibition by leflunomide or shRNAs suppressed cell proliferation, induced cell cycle arrest at S phase, as well as promoted programmed cell death including intrinsic apoptosis and autophagy in human melanoma cells. And we found the molecular mechanisms dictating cell cycle arrest and programed cell death induced by DHODH inactivation/deficiency. Our results provided clues for leflunomide and DHODH target used in malignant melanoma treatments.

## MATERIALS AND METHODS

### Cell culture

The human melanoma cells A375 were purchased from American Type Culture Collection (ATCC, Rockville, MD, USA). MV3 were purchased from the Third Military Medical University. Cell line A375 was cultured in Dulbecco's modified Eagle's medium (DMEM, Life Technologies, Grand Island, NY, USA) and MV3 was grown in Roswell Park Memorial Institute-1640 (RPMI-1640, Life Technologies, Grand Island, NY, USA), simultaneously mixed with 10% fetal bovine serum (FBS, Life Technologies, Grand Island, NY, USA) as a supplement. All cells were incubated at 37°C in humidified incubator with 5% CO2.

### Reagents

DHODH inhibitor leflunomide (Sigma), apoptotic inducer actinomycin D (Sigma) and doxorubicin (Sigma), antitumor drug temozolomide (TMZ, Sigma), 3-MA (Sigma), chloroquine (CQ, Sigma), BCL-2 inhibitor ABT-199 (Sigma) were dissolved in dimethyl sulfoxide (DMSO, Sigma). Uridine (Sigma) was dissolved in ddH_2_O. In our experiments, DMSO was used as control (untreated).

### Transfection and infection assay

The cDNA sequences of DHODH (NM 001361.4) and BCL-2 (NM 000633.2) were gained from NCBI. The RNAi candidate target sequences as follows: shDHODH#1 (AAGTGAGAGTTCTGGGCCATAAATTCC), shDHO DH#2 (AATTGCTGCAGGATTTGACAAGCATGG), shBCL-2#1 (AACCGGGAGATA GTGATGAAGTAC ATC), shBCL-2#2 (AAGTACATCCATTATAAGCTG TCGCAG). These sequences were inserted into the pLKO.1 vector. Human full length BCL-2 cDNA fragment was then cloned into PCDH-CMV-MCS-EF1-puro vector. The GFP-LC3B plasmid was a gift by Prof. Ning Gao at the Third Military Medical University, China. The shRNA plasmid with the packaging plasmids pLP1, pLP2, and VSVG (Invitrogen) mixed with Lipofectamine 2000, then this compound was transfected to 293FT cells. Afterwards virus-containing supernatants which used for cell infections were collected. After infection, the aim cells were selected in the presence of 4 μg/mL puromycin for 3 days, and drug-resistant cells were collected for the following experiment.

### MTT assay

The cell growth was of human melanoma cell lines A375and MV3 was evaluated by 3-(4,5-dimethylthiazol-2-yl)-2,5-diphenyl tetrazolium bromide (MTT, Sigma) assay. That is, about 2000 cells/well were evenly distributed in 96-well plate. After dealt with leflunomide at 50 μM, 100 μM and 200 μM concentration respectively, and DMSO was used as a control for the indicated time, 20 μl MTT was added to each well, then incubated at 37°C for 2 h. Finally, the absorbance value was measured at a wavelength of 560 nm after shaking for 10 min.

### Brdu staining assay

Cells were seeded on 24-well plate and treated with 100 μM leflunomide (and equal volume of DMSO as control) for 72 h. After that, incubated with 10 μg/ml thymidine analog 5-bromo-2 deoxyuridine (Brdu; Sigma) for 30 min at indoor temperature, then discarded the supernatant and washed thrice with PBS, anchored by 4% paraformaldehyde for 15 min, permeabilized with 0.5% Triton X-100 for 10 min after pre-treated with 2 M HCl for 10 min, blocked with 10% goat serum for 1 h, followed by incubated a monoclonal rat primary antibody against Brdu (1:300, Sigma) overnight, followed by Alexa FluorR^®^ 594 goat anti-rat IgG secondary antibody (H+L; Invitrogen). 500 μl Hoechest33342 was used for nucleus staining. In the end, the percentage of Brdu was calculated under more than 8 microscopic fields (Nikon 80i, Nikon Corporation, Tokyo, Japan).

### Cell cycle assay

Cells were plated in 60 mm plates and treated with 100 μM leflunomide (and equal volume of DMSO as control) for 72 h. Afterwards cells were washed by pre-cooling PBS, anchored by 70 % ethanol for 24 h. After that, cells was stained with propidium iodide (PI) at 37°C for 1 h in dark. Finally collected cells by the FACS C6 (BD Biosciences, San Jose, CA, USA) and data was analyzed with FlowJo6.0 software.

### Cell apoptosis assay

Cells were plated in 60 mm plates and treated with 100 μM leflunomide (and equal volume of DMSO as control) for 72 h. Afterwards cells were washed by pre-cooling PBS and collected. Cells were resuspended in 100 μl binding buffer, then stained with 5 μl propidium iodide (PI) and 5 μl Annexin V-FITC at room temperature for 20 min in dark. Finally collected cells by the FACS C6 (BD Biosciences, San Jose, CA, USA) and data was analyzed with FlowJo6.0 software.

### Western blot assay

Cells were treated with leflunomide for 72 h, then cells were collected, after that proteins were extracted by RIPA Lysis Buffer which contained 1% Phenyl methane sulfonyl fluoride (PMSF), stockpiled at −80°C. The proteins were separated by SDS-PAGE, subsequently, transfered onto PVDF membranes (Millipore, USA). The PVDF membranes were blocked with 5% BSA for 2 h, and incubated with a primary antibody against human Tubulin (1:1000, Beyotime), LC3B (1:1000, Cell Signaling), CDK2 (1:1000, Cell Signaling), CyclinA2 (1:1000, Cell Signaling), DHODH (1:1000, Cell Signaling), BCL-2 (1:1000, Cell Signaling), Caspase-9 (1:1000, Cell Signaling), Caspase-3 (1:1000, Cell Signaling), pT172-AMPK (1:800, Cell Signaling), AMPK (1:800, Cell Signaling), pSer555-Ulk (1:1000, Cell Signaling), Ulk (1:1000, Cell Signaling), pThr 183/186-JNK (1:2000, Abcam), JNK (1:1000, Abcam), BCL-2 (1:1000, Cell Signaling) and Beclin1 (1:1000, Cell Signaling) at 4°C overnight. Then incubated with homologous secondary antibodies HRP-labeled goat anti-rabbit IgG (H+L) (1:2000, Beyotime) or goat anti-mouse IgG (H+L) (1:2000, Beyotime) for 2 h. Finally, the signal was captured by the ECL reagent (Beyotime) and visualized by Western blotting detection instruments (Clinx Science).

### Immunofluorescence staining assay

Cells were seeded on cleaned and autoclaved glass slide, exposured to 100 μM Leflunomide for 72 h (DMSO as control). After that, anchored by 4% paraformaldehyde for 15 min, permeabilized with 0.5% Triton X-100 for 10 min after pre-treated with 2 M HCl for 10 min, blocked with 10% goat serum for 1 h. Then a monoclonal rat primary antibody against LC3B (1:200, Cell Signaling) vernight at 4°C. Followed by incubation with Alexa Fluor 488 goat anti-rabbit IgG (H+L) (1:2000, Beyotime), which as a secondary antibody. Then Hoechst 33342 (Sigma-Aldrich, USA) staining nuclei for 20 min. Fluorescent images were captured with a Zeiss LSM laser scanning confocal microscope.

### Co-Immunoprecipitation assay

Cell lysates was pretreated by adding 20 μl Protein A+G Agarose (Beyotime, Jiangsu, China) for 3 hours at 4°C. Precleared lysates were incubated with anti-Beclin1 antibodies (Abcam) or normal Ig G (Beyotime) overnight at 4°C. And then associated proteins with each antibody were precipitated with protein agarose A+G for 6 hours at 4°C. Immunoprecipitates were resolved by polyacrylate gel electrophoresis and analyzed by western blotting.

### Tumor xenografts assay

For tumor xenografts, 4 weeks old female nude mice (BALA/c) were purchased from Beijing laboratory animal research center. They were allowed to acclimate for 1 week in the SPF room. Cells were grown to 70–80% confluence collected and resuspended. 1 × 10^6^ cells in 100 ul DMEM or 1640 were used in subcutaneous injection. A week later, the mice were divided into two groups randomly. One group delt with leflunomide at 7.5 mg/kg, in the meantime, the other group was injected with equal volume of DMSO as a control for 12 days. Tumor growth was measured by vernier caliper every three day, then tumor volume was calculated by (small diameter)^2^ × (large diameter)/2. Finally, tumors were stripped from the mice and weighed. All animal experiments were pre-approved by the Institutional Animal Care and Use Committees of the Southwest University.

## SUPPLEMENTARY MATERIALS FIGURES AND TABLES


