# Supplementary material for: Inactivation/deficiency of DHODH induces cell cycle arrest and programed cell death in melanoma
Source: Oncotarget. 2017 Jul 19;8(68):112354–70. doi: 10.18632/oncotarget.19379 (PMC5762515; doi:10.18632/oncotarget.19379)
Supplement: Supplementary file 1 [file oncotarget-08-112354-s001.pdf]

Inactivation/deficiency of DHODH induces cell cycle arrest and programed cell death in melanoma

SUPPLEMENTARY MATERIALS

MATERIALS AND METHODS

Soft agar colony formation assay

Cells were collected and resuspended. Then 1.5 ml/well DMEM or RPMI 1640 medium containing 0.6% agarose were added to a 6-well plate and allowed to solidify (base agar). Then 1ml/well DMEM or RPMI 1640 medium containing 0.3% agarose with or without 100 μM leflunomide mixed with 1000 cells were added to the top of base agar (top agar). After incubation for 14 to 21 days. At the end, cells were stained with MTT, then imaged using a digital camera. Colonies with more than 50 cells were counted using the inverted microscope.

Quantitative real-time PCR (qRT-PCR)

qRT-PCR was conducted as previously reported [1]. The individual values were normalized to that of the *gapdh* control. Primers for Bcl-2 family members were showed as in Supplementary Table 1.

REFERENCES

1. Hu H, Dong Z, Tan P, Zhang Y, Liu L, Yang L, Liu Y, Cui H. Antibiotic drug tigecycline inhibits melanoma progression and metastasis in a p21CIP1/Waf1-dependent manner. Oncotarget. 2016; 7:3171–3185. https://doi.org/10.18632/oncotarget.6419.

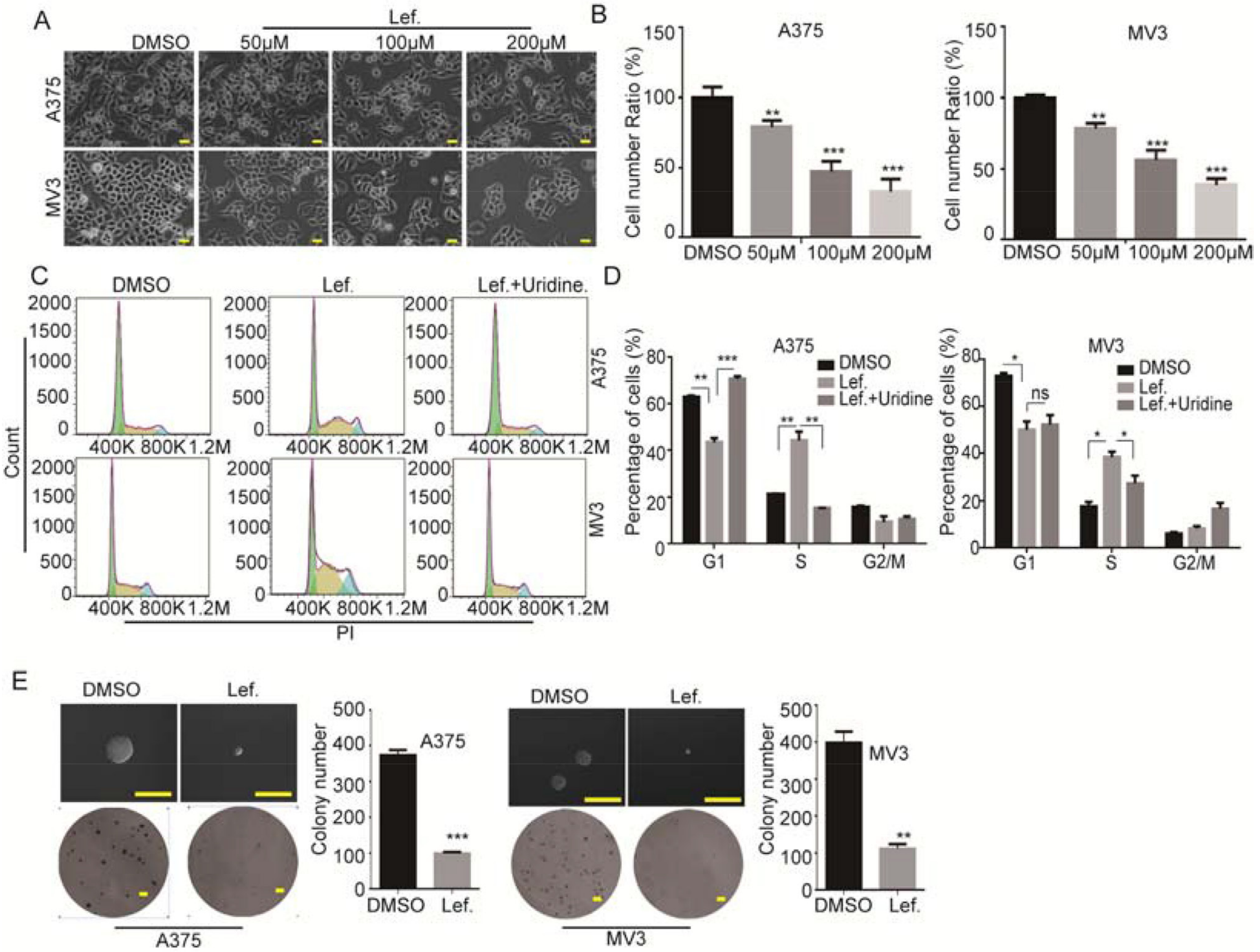

Supplementary Figure 1: DHODH inhibitor leflunomide inhibites cell proliferation and induces cell cycle arrest at S phase in melanoma cells. (**A**) The human melanoma cell line A375 and MV3 were treated with DMSO or 50 μM, 100 μM, 200 M leflunomide for 72 h respectively. Scale bar, 20 μm. (**B**) The cell number of leflunomide-treated group was calculated and normalized by that in DMSO-treated group. (**C** and **D**) Cell cycle was analyzed by flow cytometry in DMSO or 100 μM leflunomide-treated A375 and MV3 cells in the presence and absence of 1 mM uridine in culture medium for 72 h. (**E**) Soft agar assays were performed after treated with DMSO, 100 μmol/L leflunomide for 72 h. The quantification of colony numbers was also presented. Scale bars, 200 μm. All data were shown as the mean ± SD, Student's *t*-test was carried out. $^{*}p < 0.05$,$^{**}p < 0.01$, $^{***}p < 0.001$.

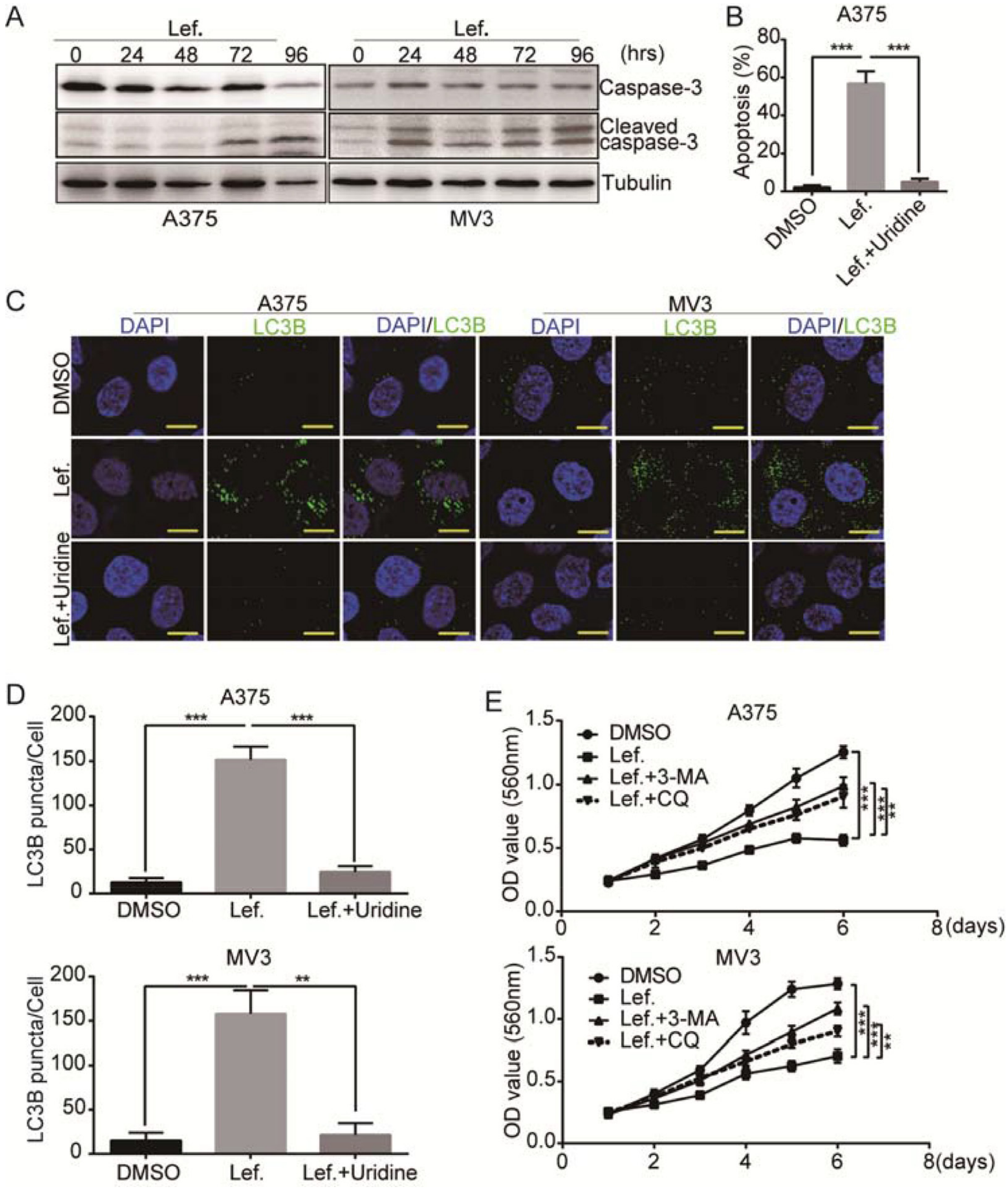

Supplementary Figure 2: Leflunomide induces apoptosis and autophagy in melanoma cells. (**A**) Western blot assay was performed to assess the cell apoptosis-related protein levels in DMSO or 100 μM leflunomide-treated A375 cells in the presence and absence of 1mM uridine in culture medium. (**B**) The rate of apoptosis was analyzed using flow cytometry in A375 cells after DMSO or 100 μM leflunomide treatment in the presence and absence of 1 mM uridine in culture medium. (**C** and **D**) Immunofluorescence staining with a LC3B antibody was performed to confirm the induction of autophagy in DMSO or 100 μM leflunomide-treated A375 and MV3 cells in the presence and absence of 1 mM uridine in culture medium for 72 h. Representative LC3B-positive cells are shown. Scale bars, 10 μm. (**E**) Cell growth was tested by the MTT assay in DMSO or 100 μM leflunomide-treated A375 and MV3 cells in the presence and absence of 3-MA (10 mM) and chloroquine (CQ, 25 μM) in culture medium for 1–6 days. All data were shown as the mean ± SD, Student's *t*-test was carried out. $^{**}p < 0.01$, $^{***}p < 0.001$.

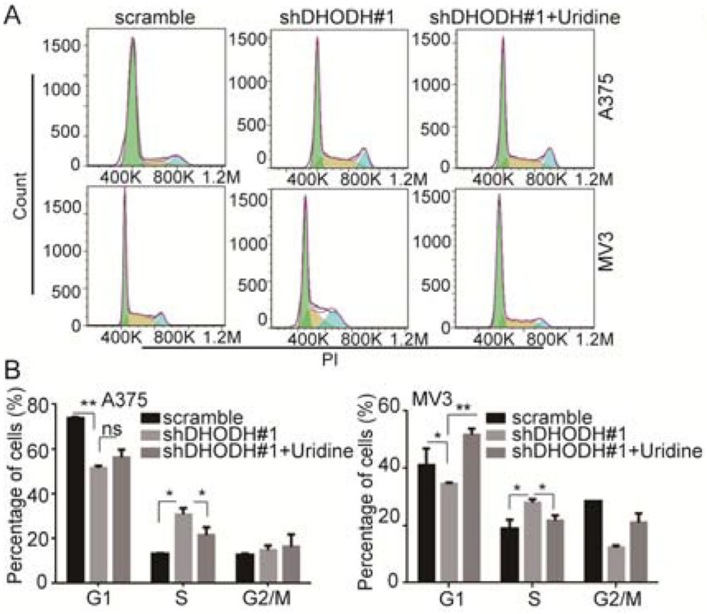

Supplementary Figure 3: DHODH knockdown suppresses cell proliferation and induces cell cycle arrest at S phase in melanoma cells. (**A** and **B**) The cell cycle was analyzed by flow cytometry in DHODH knockdown A375 and MV3 cells in the presence and absence of 1mM uridine in culture medium. (**C**) Soft agar assays were performed after DHODH knockdown. The quantification of colony numbers was also presented. Scale bars, 200 μm. All data were shown as the mean ± SD, Student's *t*-test was carried out. $^{*}p < 0.05$, $^{**}p < 0.01$.

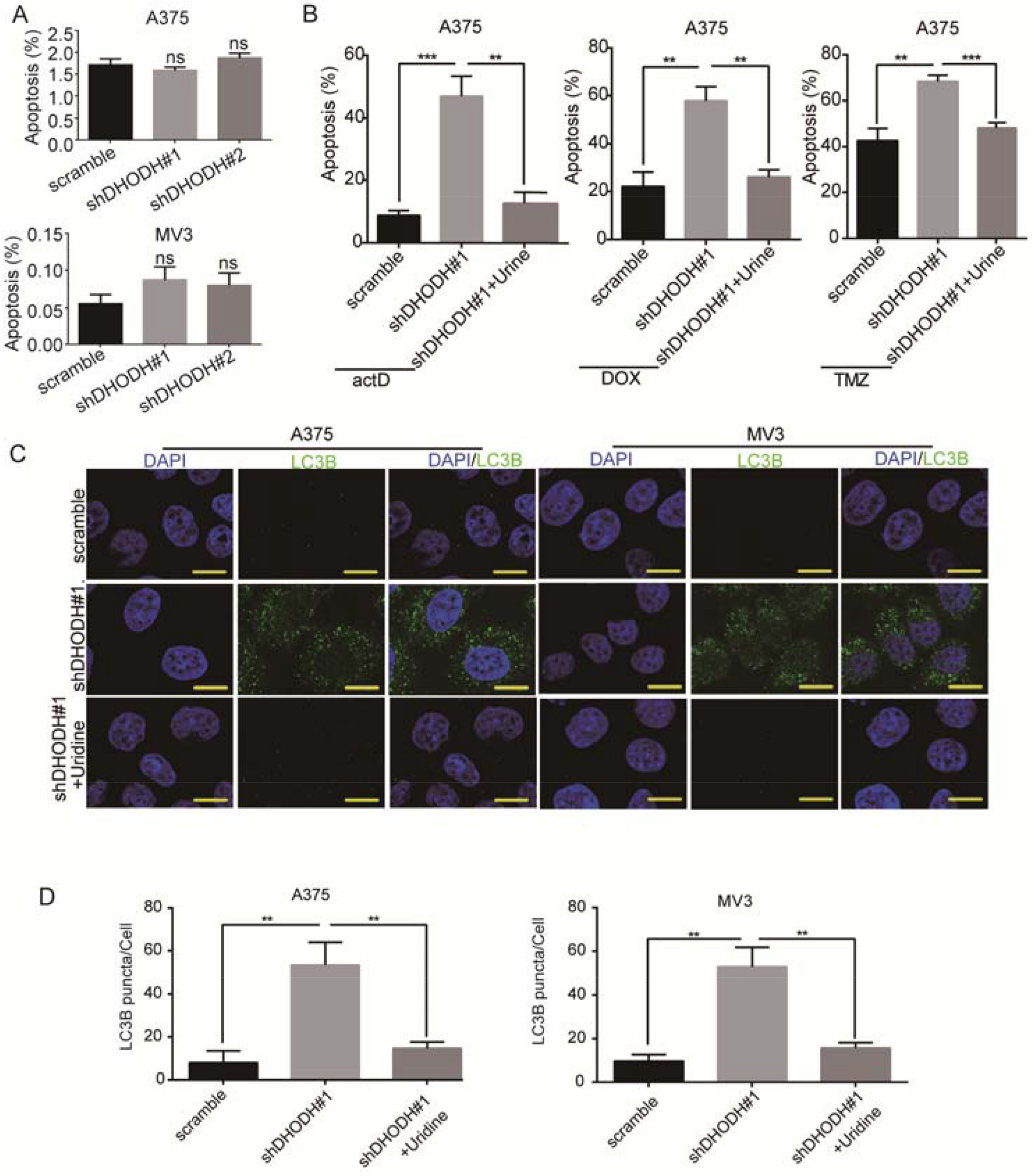

Supplementary Figure 4: DHODH downregulation sensitizes drug-induced apoptosis and induces autophagy in melanoma cells. (**A**) The rate of apoptosis of A375 and MV3 cells was analyzed using flow cytometry after knock down of DHODH. (**B**) The rate of apoptosis was analyzed using flow cytometry in DHODH-knockdown A375 cells after 1mM uridine treatment in the presence of 1 nM actinomycin D (ActD) for 24 h, 0.5 μM doxorubicin (DOX) for 12 h, or 400 μM temozolomide (TMZ) for 72 h in culture medium. (**C** and **D**) Immunofluorescence staining with a LC3B antibody was performed to confirm the induction of autophagy in DHODH-knockdown A375 and MV3 cells after 1 mM uridine treatment for 72 hours. Representative LC3B-positive cells are shown. Scale bars, 10 μm. All data were shown as the mean ± SD, Student's *t*-test was carried out. $^{**}p < 0.01$, $^{***}p < 0.001$, ns, no sense.

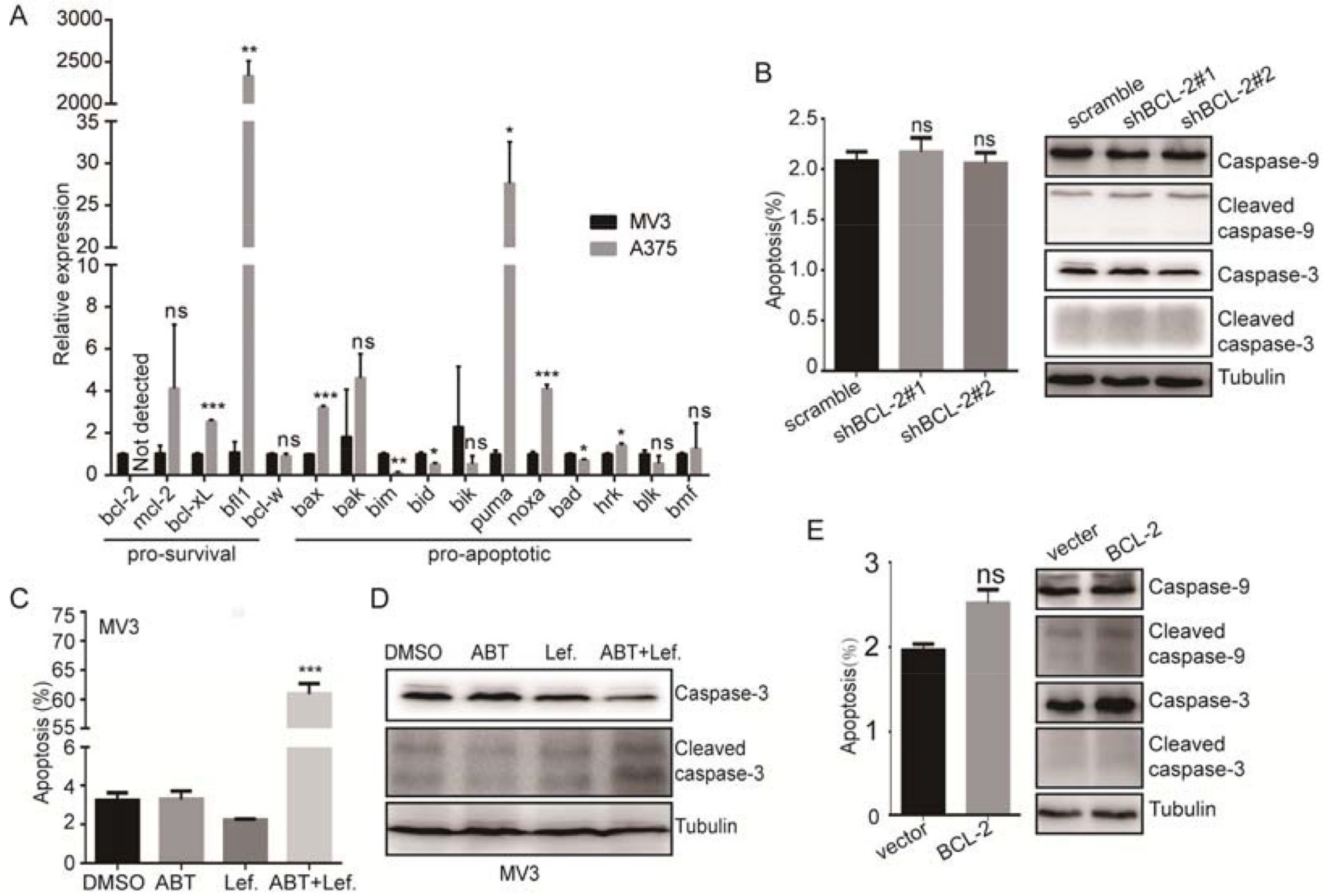

Supplementary Figure 5: BCL-2 is a switch of apoptosis induced by DHODH inhibition. (**A**) RT-PCR analysis of the different expression of BCL-2 family members between MV3 and A375 cells. *Gapdh* expression was used as control. (**B**) The rate of apoptosis of MV3 cells after BCL-2 knockdown was analyzed using flow cytometry. (**C**) The rate of apoptosis of MV3 cells was analyzed using flow cytometry after treated with leflunomide with or without 20 μM ABT-199 (ABT) for 72 h. (**D**) Western blot assay was performed to assess the cell apoptosis-related protein levels in MV3 cells after treated with 100 μM leflunomide with or without 20 μM ABT-199 for 72 h. (**E**) The rate of apoptosis of MV3 cells after BCL-2 overexpressed was analyzed using flow cytometry. All data were shown as the mean ± SD, Student's *t*-test was carried out. $^{*}p < 0.05$, $^{**}p < 0.01$, $^{***}p < 0.001$, ns, no sense.

Supplementary Table 1: Primers for Bcl-2 family members

Target	Forward	Reverse
bcl-2	CCCTGTGGATGACTGAGTACC	TGAGCAGAGTCTTCAGAGACAG
mcl-2	GCCTTCCAAGGATGGGTTTG	AGGTTGCTAGGGTGCAACTC
bcl-xL	TTCCGGGATGGGGTAAACTG	ACAAAAGTATCCCAGCCGCC
bfl1	AAATTGCCCCGGATGTGGAT	ACAAAGCCATTTTCCCAGCCT
bcl-w	CCTTCTTTGTCTTTGGGGCTG	GTATAGAGCTGTGAACTCCGC
bax	CCCAGAGGCGGGGTTTCAT	AGCTGCCACTCGGAAAAAGA
bak	GAGGATCTACAGGGGACAAGT	GCCCCGAAGCCATTTTTCAG
bim	AGACAGAGCCACAAGGTATTTT	GTATCTCGGCTCCGCAAAGA
bid	CCACACCGTGGTCTTTCCA	GCACATCATTGCCAGTGCTC
bik	GATGCCTTTTATATTAACCCCGTG	CCGAGGGCATCACATATCACA
puma	CCACGGCTTTGGAAAAAGGA	CTCCCTGGGGCCACAAATCT
noxa	ATTACCGCTGGCCTACTGTG	ATGTGCTGAGTTGGCACTGA
bad	CGGAGGATGAGTGACGAGTT	CCAAGTTCCGATCCCACCAG
hrk	AGCAACAGGTTGGTGAAAACC	TTTCTACGATCGCTCCAGGC
blk	ACCCCGAGATAGTGCTGGAA	TATCACACAGGGGCGTACCA
bmf	TATTTTGGAAACAATACCGCACCG	CCAGACTCGATTGGGAAGGA
gapdh	TATAAATTGAGCCCGCAGCC	GTTTCTCTCCGCCCGTCTT
